# Intralabyrinthine Schwannoma: Distinct Features for Differential Diagnosis

**DOI:** 10.3389/fneur.2019.00750

**Published:** 2019-07-23

**Authors:** Sun-Uk Lee, Yun Jung Bae, Hyo-Jung Kim, Jeong-Yoon Choi, Jae-Jin Song, Byung Yoon Choi, Byung-Se Choi, Ja-Won Koo, Ji-Soo Kim

**Affiliations:** ^1^Department of Neurology, Korea University Anam Hospital, Seoul, South Korea; ^2^Department of Neurology, Seoul National University College of Medicine, Seoul, South Korea; ^3^Department of Radiology, Seoul National University Bundang Hospital, Seongnam-si, South Korea; ^4^Research Administration Team, Seoul National University Bundang Hospital, Seongnam-si, South Korea; ^5^Dizziness Center, Clinical Neuroscience Center, and Department of Neurology, Seoul National University Bundang Hospital, Seongnam-si, South Korea; ^6^Department of Otolaryngology-Head and Neck Surgery, Seoul National University College of Medicine, Seoul National University Bundang Hospital, Seongnam-si, South Korea

**Keywords:** vertigo, nystagmus, meniere's disease, vestibular schwannoma, vestibulo-ocular reflex, head-impulse test

## Abstract

**Objectives:** The aim of this study was to delineate the clinical and laboratory features suggestive of intralabyrinthine schwannoma (ILS).

**Methods:** We compared the clinical features of 16 patients with ILS, who had been diagnosed at the Seoul National University Bundang Hospital from 2003 to 2018, with those of 18 patients with symptomatic unilateral intracanalicular schwannoma and randomly selected 20 patients with definite or probable unilateral Meniere's disease (MD).

**Results:** Patients with ILS presented with either recurrent spontaneous dizziness/vertigo combined with auditory symptoms (*n* = 8), isolated auditory symptoms without dizziness/vertigo (*n* = 7), or recurrent spontaneous dizziness/vertigo without auditory symptoms (*n* = 1). Most patients reported no improvement (*n* = 11) or worsening (*n* = 1) of the symptoms despite medical treatments including intratympanic (*n* = 5) or intravenous steroids (*n* = 2). Conventional brain MRIs failed to detect ILS in about a half of the patients (7/16, 44%). However, ILS showed a filling defect on 3-dimensional (3D) heavily T2-weighted MRIs (*n* = 12), and nodular enhancement on 3D contrast-enhanced T1 (*n* = 15) or FLAIR MRIs (*n* = 13) targeted for the inner ear. Compared to MD or intracanalicular schwannoma, ILS showed mostly abnormal head-impulse tests (HITs, *p* = 0.001). In contrast, the incidence of canal paresis did not differ among the groups (*p* = 0.513).

**Conclusion:** ILS may mimic MD by presenting recurrent dizziness/vertigo and auditory symptoms. ILS should be suspected in patients with recurrent audiovestibulopathy especially when (1) the duration of the dizziness is not typical for MD, (2) the patients do not respond to medical treatments, or (3) HITs are abnormal.

## Introduction

Vestibular schwannoma is one of the common benign tumors that arise from the vestibulocochlear nerve. It is termed intralabyrinthine schwannoma (ILS) when the tumor is originated from the Schwann cells surrounding the terminal branch of the vestibulocochlear nerve inside the membranous labyrinth (1, 2). ILS may mimic Meniere's disease (MD) or inflammatory disorders involving the inner ear by presenting recurrent dizziness/vertigo and fluctuating or progressive tinnitus, ear fullness and hearing loss (3, 4). Indeed, ILS was once found during labyrinthectomy in a patient with a preoperative diagnosis of intractable MD (5). Otherwise, ILS may be an incidental finding at autopsy (5, 6). Slow growth of ILS also contributes to delayed diagnosis of the tumor (7). Furthermore, routine brain MRIs, even with gadolinium enhancement, frequently fail to reveal ILS. Thus, antemortem diagnosis of ILS remains challenging unless sought with a strong suspicion (4).

Several anecdotal studies have described the neurotologic findings in ILS, which include ipsilesional canal paresis (4, 6, 8, 9) abnormal cervical or ocular vestibular-evoked myogenic potentials (VEMPs) (6, 10), and low-tone hearing loss (4). However, systematic analyses of clinical and laboratory neurotologic findings have not been available in ILS. This study aimed to compare the clinical features and to delineate the findings suggestive of ILS among the disorders presenting recurrent or progressive audiovestibulopathy, especially in comparison with MD and intracanalicular schwannoma.

## Materials and Methods

### Subjects

**Figure 1:**
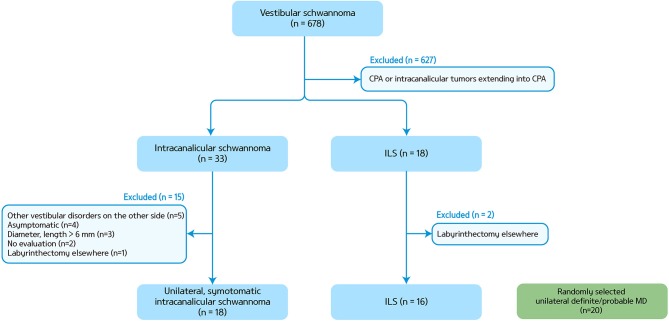
A flow chart for patient selection. CPA, cerebellopontine angle; ILS, intralabyrinthine schwannoma; MD, Meniere's disease.

We retrospectively reviewed the medical records of 678 patients who had the diagnosis of vestibular schwannoma at Seoul National University Bundang Hospital from July 2003 to July 2018. Among them, we were able to find 16 patients with ILS (eight men, mean age ± SD = 49 ± 14, Figure 1). All patients showed a unilateral lesion (nine on the right side). The diagnosis of ILS was made with MRIs in 15 patients according to the criteria described previously (11), and with pathology in the remaining one (patient 2). Ten patients had been regularly followed up for more than a year at the outpatient clinic until the study, and the other six patients provided additional information on the clinical course over the telephone.

We also analyzed the findings of 18 patients with symptomatic unilateral intracanalicular schwannoma of which the length and diameter were <6 mm (seven on the right side, seven men, mean age ± SD = 65 ± 14) and randomly selected 20 patients with definite/probable unilateral MD (eight on the right side, seven men, mean age ± SD = 47 ± 17) (12). Intracanalicular schwannoma was defined when the tumors reside in the internal acoustic canal and project no more than 2 mm from the internal acoustic meatus.

This study followed the tenets of the Declaration of Helsinki and was reviewed and approved by the Institutional Review Board of Seoul National University Bundang Hospital (B-1112/141-003).

### Neurotologic Evaluations

In addition to standard neurologic examination, patients also had evaluation of spontaneous, gaze-evoked (GEN), vibration-induced, head-shaking and positional nystagmus, and HITs using video-oculography (13–15).

Patients also had bithermal caloric tests, and measurements of ocular torsion and subjective visual vertical (SVV), and cervical and ocular VEMPs. Detailed methods of each test have been described previously (16, 17).

Pure-tone thresholds were obtained at frequencies of 0.25, 0.5, 1, 2, 3, 4, and 8 kHz with calibrated pure-tone audiometry in a sound proof audio booth. The pure-tone average was calculated as an average of the pure-tone thresholds measured at 0.5, 1, 2, and 3 kHz (AMA guides, 6th ed.).

### MRI Protocol

MRI was performed on a 3T scanner (Achieva and Ingenia; Philips Healthcare, Best, The Netherlands) with a 16- or 32-channel SENSE Head Coil (Philips Healthcare). The MRI protocol included diffusion-weighted imaging (DWI), T1-, and T2-weighted gradient echo axial imaging, and T1-weighted sagittal imaging.

To delineate the structures within the labyrinth, additional sequences were applied to include 3D heavily T2- (T2-weighted volume isotropic turbo spine-echo acquisition, VISTA), 3D contrast-enhanced T1, and 3D contrast-enhanced fluid-attenuated inversion recovery (FLAIR) images in all patients. In addition, when the differentiation between ILS and labyrinthitis was not evident, a quantitative analysis of the signal intensity was performed for detection of ILS by two neuroradiologists who were blinded to the clinical findings. ILS was diagnosed radiologically when the lesion showed enhancement on 3D contrast-enhanced T1-weighted or 3D contrast-enhanced FLAIR images, in combination with a filling defect and replacement of the normal high-signal intensity fluid on 3D VISTA (1, 11). The detailed methods for imaging and analysis protocols were described elsewhere (11). We also classified ILS according to the tumor location in the labyrinth using the previously described methods (1, 18).

### Statistical Analyses

Statistical analyses were also performed using SPSS (version 18.0; SPSS, Chicago, IL, USA). Continuous variables were compared using Mann-Whitney test or ANOVA with a Bonferroni correction, and nominal variables were compared using χ^2^ or Fisher's exact test. Correlation analysis was conducted using Spearman correlation. The significance level was set at *p* < 0.05.

## Results

### Clinical Features of ILS

Patients with ILS usually presented recurrent spontaneous dizziness/vertigo and auditory symptoms, mimicking MD (*n* = 8, Table 1). Otherwise, they showed isolated auditory symptoms without dizziness or vertigo (*n* = 7), or recurrent spontaneous dizziness/vertigo without auditory symptoms (*n* = 1). The dizziness/vertigo lasted from a few seconds up to a day. One patient (patient 2) also experienced recurrent Tumarkin attacks. The hearing loss was uniformly progressive after initial fluctuation (14/16, 88%). Four patients also experienced sudden hearing loss (4/16, 25%, Table 1). Among those with combined audiovestibulopathy, the onsets of vestibular and audiologic symptoms were sequentially spaced by 6 months to 10 years (Table 1). The vestibular symptoms preceded the audiologic symptoms in five and vice versa in the other three.

**Table 1:** Clinical and neurotologic findings in the patients with intralabyrinthine schwannoma.

**Pt**	**Age**	**Lesion side**	**Vestibular symptom**			**Auditory symptom**				**Neurotologic findings**				
			**Onset-to-presentation**	**Duration**	**Frequency**	**Onset-to-presentation**	**Tinnitus**	**EF**	**HL**	**SN**	**HSN**	**HIT**	**Caloric test (CP, %)**	**Pure tone average (dB)**
**COMBINED AUDIOVESTIBULAR SYNDROME**														
1	50–55	L	1 year	5 min	3 ~ 4/year	−1 month^§^	L	L	L	–	–	?	?	36
2	60–65	R	10 years	Few seconds ~ 2 h^††^	2 ~ 3/month	9 months	R	R	R^*^	–	**I**	**I**	**R (77)**^†^	68
3	40–45	L	9 years	12 h	1/week	8 months	–	–	L	–	**C**	Normal	**L (68)**	68
4	40–45	L	4 years	Few seconds	1/2 years	6 years	L	–	L	**C**	**C**	**I (HC, AC, PC)**	**L (100)**	68
5	45–50	R	16 years	24 h	3 ~ 4/month	5.5 years	R	R	R	–	–	**B (HC)**	?	68
6	55–60	R	3 years	2 ~ 3 h	1/year	11 years	R	R	R^*^	**I →** **C**	**C**	**I (HC, AC, PC)**	**R (94)**	68
7	60–65	L	2 years	Few seconds	3 ~ 4/day	−4.5 years^§^	–	L	L	–	–	Normal	L (11)	51
8	15–20	R	1 year	6 h	2 /month	1.5 years	R	R	R	–	**C**	**I (HC, AC, PC)**	**R (85)**	68
**ISOLATED VESTIBULAR SYNDROME**														
9	70–75	R	11 years	Few seconds	3 ~ 4/year		–	–	B	–	**C**	**I (HC, AC, PC)**	**R (53)**	56
**ISOLATED AUDITORY SYNDROME**														
10	45–50	L				1.5 years	L	L	L	**C**	–	**I (HC, PC)**	**L (100)**	66
11	50–55	R				1 month	R	–	R	?	?	**I**^†^	?	52
12	35–40	L				2 months	L	L	L	–	–	?	?	20
13	55–60	L				1 month	L	–	L^*^	–	–	Normal	R (17)	68
14	60–65	R				2 months	R	R	R^*^	–	–	Normal	?	24
15	45–50	R				14 years	–	–	R	?	?	**I**^†^	**R (90)**	63
16	25–30	R				2 years	–	–	R^*^	–	–	Normal	L (15)	29

* *This patient presented sudden hearing loss*.

† *Head-impulse tests were done on bedside*.

†† *This patients also experienced recurrent Tumarkin attacks*.

§ *The symptom started during the follow-up. AC, anterior canal; B, bilateral; C, contralesional; CP, canal paresis; HC, horizontal canal; HIT, head-impulse test; HL, hearing loss; HSN, head-shaking nystagmus; I, ipsilesional; L, left; PC, posterior canal; R, right; SN, spontaneous nystagmus*.

*Abnormal results are presented as bold*.

Patients' symptoms did not respond to intratympanic (*n* = 4) or intravenous steroids (*n* = 2) in six of seven patients with administration. Nine patients were subjected to medication regularly or as necessary with a follow-up for at least 6 months, which included betahistine (*n* = 4, 12 ~ 36 mg a day), diazepam (*n* = 3, 4 mg a day), cinnarizine/dimenhydrinate (*n* = 3, 40 mg/80 mg a day), isosorbide (*n* = 3, 15 ~ 90 ml a day), Ginko Biloba extract (*n* = 2, 80 mg a day), nortriptyline (*n* = 1, 10 mg a day), carbamazepine (*n* = 1, 400 mg a day), in various combination. During the follow-up of 2 month to 6 years (median = 1 year) from the initial presentation, most patients reported no improvement (*n* = 11) or worsening (*n* = 1, patient 7) of the symptoms, while the other four reported an improvement. Three patients [3/16 (19%), patients 2, 4, and 8] with severe and intractable symptoms, including one with a tumor growth on follow-up MRIs, underwent a labyrinthectomy.

### Clinical Features of Intracanalicular Schwannoma

The patients with intracanalicular schwannoma also presented recurrent spontaneous dizziness/vertigo with (*n* = 11) or without auditory symptoms (*n* = 6) except one with a sudden hearing loss (*n* = 1). The dizziness/vertigo spell lasted from a few seconds to 3 days and occurred from once every 4 years to nearly daily. Most patients remained stable, but dizziness or hearing loss worsened in three despite intravenous steroids (*n* = 3). Besides, four (4/15, 27%) patients showed a tumor growth on the follow-up MRIs at least 1 year later, and two of them underwent a gamma-knife surgery.

### Neurotologic Findings of ILS

The neurotologic findings of the patients with ILS are summarized in Table 1. Twelve patients were evaluated between the attacks except one (patient 6). Spontaneous nystagmus beating to the healthy ear was observed in three patients (3/14, 21%). Head-shaking (6/14, 43%) and vibration-induced nystagmus (7/13, 54%) were observed in about a half of patients and were mostly contralesionally beating when observed. Positional changes did not evoke in nine patients tested.

Cervical VEMPs were mostly abnormal (8/11, 77%) either during stimulation of the ear on the lesion side (*n* = 7, absent responses in six and decreased responses in the other one), or both sides (*n* = 1). Similarly, ocular VEMPs were mostly abnormal during stimulation of the affected ear (5/8, 63%). SVV was tilted to the lesion side in two of the four patients tested. No patient showed skew deviation or abnormal ocular torsion.

**Figure 2:**
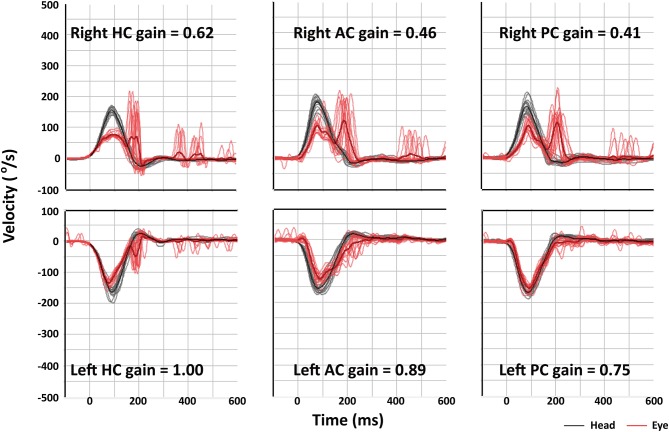
Video head-impulse tests (HITs) of patient 9 shows decreased vestibulo-ocular reflex (VOR) gains and corrective saccades for all three semicircular canals on the right side. AC, anterior canal; HC, horizontal canal; PC, posterior canal.

Compared to those with MD or intracanalicular schwannoma, HITs were more frequently abnormal in ILS patients [2/20 (10%) vs. 2/15 (13%) vs. 9/14 (64%), *p* = 0.001, Table 2, Figure 2). Abnormal video HITs were found in 55% (6/11) for the horizontal, 36% (4/11) for the anterior, and 45% (5/11) for the posterior canals after excluding three patients with bedside HITs only. However, the incidence of canal paresis did not differ among the groups [11/19 (58%) vs. 7/14 (50%) vs. 8/11 (73%) *p* = 0.513).

**Table 2:** Neurotologic findings in patients intralabyrinthine schwannoma (ILS) and Meniere's disease (MD).

	**ILS (*n* = 16)**	**MD (*n* = 20)**	**Intracanalicular schwannoma (*n* = 18)**	***p*-value**
Age, mean ± SD, years	49 ± 14	47 ± 17	65 ± 14	**0.002,** 0.012^*^
Sex, men (%)	8/16 (50)	7/20 (35)	8/18 (44)	0.652
Spontaneous nystagmus (%)	3/14 (22)	2/20 (10)	2/17 (12)	0.609
Abnormal HITs (%)	9/14 (64)	2/20 (10)	2/15 (13)	**0.001**
Canal paresis (%)	8/11 (73)	11/19 (58)	7/14 (50)	0.513
Head-shaking nystagmus (%)	6/14 (43)	7/19 (37)	3/17 (18)	0.276
Vibration-induced nystagmus (%)	7/13 (54)	5/15 (33)	6/15 (40)	0.539
Hyperventilation-induced nystagmus (%)	3/8 (38)	0/4 (0)	3/5 (60)	0.473
Abnormal cVEMPs (%)	8/11 (73)	12/17 (71)	8/14 (57)	0.647
Abnormal oVEMPs (%)	5/8 (63)	5/10 (50)	1/8 (13)	0.106
Hearing fluctuation on audiometry (%)	1/11 (9)	6/15 (40)	0/15 (0)	**0.010**

*cVEMPs, cervical vestibular-evoked myogenic potentials; HITs, head-impulse tests; oVEMPs, ocular VEMPs; SD, standard deviation*.

* *p-value for MD vs. intracanalicular schwannoma equals 0.002. p-value for ILS vs. MD equals 0.012*.

*Abnormal results are presented as bold*.

### MRI Findings of ILS

Conventional brain MRIs failed to detect ILS in almost half of the patients (7/16, 44%), but could detect intracanalicular schwannoma in most of the patients (13/14, 93%, *p* = 0.024).

**Figure 3:**
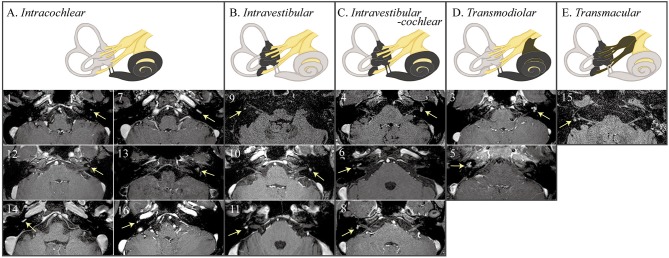
MRIs of the patients with intralabyrinthine schwannoma (ILS) according to Kennedy classification. 3D contrast-enhanced T1 or fluid attenuated inversion recovery (FLAIR) images show nodular enhancements within the labyrinth (arrows).

On 3D heavily T2-weighted images, the high-signal labyrinthine fluid was replaced by a filling defect (*n* = 12) in ILS. ILS showed nodular enhancement on 3D contrast-enhanced T1 (*n* = 15) or 3D contrast-enhanced FLAIR images (*n* = 13, Figure 3). A growth of the tumor was found in one (1/8, 13%, patient 8) of the eight patients who had a follow-up MRI at least 1 year later. ILS was classified into intracochlear in six, intravestibular in three, intravestibular-cochlear in three, transmodiolar in two, and transmacular in one patient (Figure 2).

### Neurotologic-Anatomical Correlation

Including one with intralabyrinthine-cochlear ILS documented by labyrinthectomy (patient 2), neurotologic-anatomical correlation was conducted. Patients with vestibular involvement (*n* = 9, four with intravestibular-cochlear, three with intravestibular, and one with transmacular) mostly showed abnormal HITs [8/9 (89%), Spearman correlation coefficient = 0.689, *p* = 0.006) or canal paresis [8/8 (100%), Spearman correlation coefficient = 1.0, *p* = 0.01]. However, pure tone average showed no difference between those with (*n* = 11; six with intracochlear, four with intravestibular-cochlear, and two with trans modiolar) and without (*n* = 5) cochlear involvement [median (interquartile range) = 68 (29–68) vs. 63 (56–66), *p* = 0.953].

## Discussion

Intralabyrinthine schwannoma and intracanalicular schwannoma may present combined audiovestibular syndrome, or vestibular or auditory symptoms in isolation. The onsets of vestibular and auditory symptoms were sequentially spaced by up to 10 years. Intermittent spontaneous dizziness/vertigo mostly lasts a few seconds to a day. Compared to those with MD or intracanalicular schwannoma, the patients with ILS mostly showed abnormal HITs. The abnormal caloric and HITs correlated with vestibular involvements documented on MRIs. ILS often escapes detection on conventional brain MRIs only and requires an additional imaging targeted for the inner ear.

Hearing loss has been almost always reported in ILS (5, 6, 18). It is mostly sudden and progressive, but some patients may report fluctuating or episodic hearing loss initially. Thus, the episodic dizziness/vertigo in association with fluctuating tinnitus and ear fullness in ILS may masquerade MD (3, 4, 18). Indeed, 39% of ILS patients carried a prior diagnosis of MD (18). As such, differentiation between the two disorders may be difficult based on the clinical features only.

To the best of our knowledge, this is the first study that systematically analyzed the neurotologic findings in patients with ILS. Our study implicates that neurotologic findings can aid in early detection of ILS. Previously, selective impairment of low but not higher frequency VOR has been reported in a patient with ILS (9). That is, the caloric responses were decreased while HITs were normal. It was explained by a frequency dependence of the vestibular injury or compensation. A similar dissociation between HITs and caloric tests are frequently observed in MD (19). It has been ascribed to predominant loss of type II hair cells in MD (20), or dissipation of the hydrostatic force due to endolymphatic hydrops (21, 22). In contrast, our study showed that patients with ILS mostly show abnormal results on both HITs and caloric tests (21, 22). Of interest, abnormal caloric and HITs were also observed among those who did not report any vestibular symptoms in this study. We suspect this dormant vestibulopathy may be owing to the central compensation after chronic vestibular injury (23). Otherwise, a space-occupying lesion in the labyrinth may impair the endolymphatic flow and result in abnormal HITs or canal paresis. Our study again emphasizes the importance of complete vestibular assessment even in patients with isolated auditory symptoms.

The results of current study are consistent with previous observation of canal paresis in nearly three-quarters of the patients with ILS (6). Otherwise, positional nystagmus was associated with hearing loss in ILS (24). Most patients with ILS also reported worsening of symptoms during positional maneuvers, which was explained by a mass effect on the vestibular organs, or by formation of secondary hydrops (24). In addition, abnormal ocular and cervical VEMPs can be frequently observed in intravestibular ILS (6, 10). Our study also found abnormal cervical or ocular VEMPs in nearly two-third of the patients.

The size of tumor in the cerebellopontine angle tends to inversely correlate with the head impulse gain of the VOR (25). However, our patients with intracanalicular schwannoma mostly shows normal HITs. Given the diameter of IAC (~6 mm) (26), the vestibular nerve may have been spared to display normal HITs in our patients with small intracanalicular schwannoma. Our study implicates that abnormal HITs in patients presenting recurrent audiovestibular syndrome indicate ILS or a large tumor in the cerebellopontine angle.

We were able to observe the neurotologic findings during an acute episode of vestibular attack in one patient (patient 6), and to document evolution of spontaneous nystagmus. This pattern of nystagmus evolution is frequently seen in MD during the acute phase (27). In MD, direction-changes of spontaneous nystagmus have been explained by excitation of the affected vestibular nerve (irritative nystagmus), paresis of the affected vestibular nerve after repetitive firing (paretic nystagmus), and following central compensation (recovery nystagmus) (27). In our patient, the intervening spontaneous nystagmus beating to the lesion side may have been due to transient irritation of the vestibular nerve from partial demyelination due to schwannoma, or due to temporary central compensation. Otherwise, endolymphatic hydrops might have occurred secondary to expansion of the schwannoma given that a previous histopathologic examination showed a compression of the endolymphatic duct directly by ILS and resultant endolymphatic hydrops in the vestibule or cochlea (28). Even though most patients did not respond to medical treatments, a small number of patients reported improvement of their symptoms, which may be explained by resolution of secondary endolymphatic hydrops having caused the symptoms.

Before the advent of high-resolution MRIs, the diagnosis of ILS mostly relied on autopsy or surgical pathology. However, introduction of higher field magnets and refined MRI sequences has allowed an antemortem diagnosis of ILS (1). Gadolinium-enhancement of the labyrinth may also be observed in inflammatory disorders involving the labyrinth (4). However, a filling defect indicative of a soft tissue mass inside the labyrinth should be atypical for these disorders (1, 11). Besides, a quantitative analysis of the signal intensity allows differentiation of ILS from labyrinthitis (11).

Despite a few anecdotal reports (29, 30), the safety and efficacy of gamma-knife surgery for ILS remain to be determined. Radiosurgery emerges as the best option for small tumors especially <30 mm (31). Indeed, two of our patients underwent gamma-knife surgery without a discernable complication. However, targeted radiation of the labyrinth is difficult and optimal radiation dosage is still undetermined. Besides, it can affect hearing function or fail to control tumor growth or symptoms (5).

This is the first study that attempted to determine the neurotological features of ILS. However, the sample size is rather small, and the retrospective nature of the study could not provide comprehensive neurotological evaluation, such as cervical or ocular VEMPs, in every patient.

## Conclusion

ILS may escape diagnosis unless strongly suspected in patients with fluctuating or progressive auditory symptoms and recurrent dizziness/vertigo. ILS should be considered in differential diagnosis especially when (1) the duration of the dizziness is not typical for MD, (2) the patients do not respond to medical treatments or intratympanic steroid injection, or (3) the patients with the features of MD show abnormal results on HITs. A detailed history and scrutinized evaluation of the inner ear function may allow identification of this rare but important cause of recurrent audiovestibular syndrome.

## Data Availability

The datasets generated for this study are available on request to the corresponding author.

## Ethics Statement

This study followed the tenets of the Declaration of Helsinki and was performed according to the guidelines of Institutional Review Board of Seoul National University Bundang Hospital (B-1112/141-003).

## Author Contributions

S-UL and YB analyzed and interpreted the data and wrote the manuscript. H-JK, J-YC, J-JS, BC, and B-SC analyzed and interpreted the data and revised the manuscript. J-SK and J-WK designed and conceptualized the study, interpreted the data, and revised the manuscript.

### Conflict of Interest Statement

J-WK serves on the editorial boards of the Journal of Vestibular Research and Auris Nasus Larynx. J-SK serves as an associate editor of Frontiers in Neuro-otology and on the editorial boards of the Journal of Clinical Neurology, Frontiers in Neuro-ophthalmology, Journal of Neuro-ophthalmology, Journal of Vestibular Research, Journal of Neurology, and Medicine. The remaining authors declare that the research was conducted in the absence of any commercial or financial relationships that could be construed as a potential conflict of interest.
